# The Hearing Outcomes of Cochlear Implantation in Waardenburg Syndrome

**DOI:** 10.1155/2016/2854736

**Published:** 2016-06-08

**Authors:** Hajime Koyama, Akinori Kashio, Aki Sakata, Katsuhiro Tsutsumiuchi, Yu Matsumoto, Shotaro Karino, Akinobu Kakigi, Shinichi Iwasaki, Tatsuya Yamasoba

**Affiliations:** ^1^Department of Otolaryngology, Head and Neck Surgery, Toranomon Hospital, Tokyo 105-8470, Japan; ^2^Department of Otolaryngology, Faculty of Medicine, University of Tokyo, Tokyo 113-8655, Japan

## Abstract

*Objectives*. This study aimed to determine the feasibility of cochlear implantation for sensorineural hearing loss in patients with Waardenburg syndrome.* Method*. A retrospective chart review was performed on patients who underwent cochlear implantation at the University of Tokyo Hospital. Clinical classification, genetic mutation, clinical course, preoperative hearing threshold, high-resolution computed tomography of the temporal bone, and postoperative hearing outcome were assessed.* Result*. Five children with Waardenburg syndrome underwent cochlear implantation. The average age at implantation was 2 years 11 months (ranging from 1 year 9 months to 6 years 3 months). Four patients had congenital profound hearing loss and one patient had progressive hearing loss. Two patients had an inner ear malformation of cochlear incomplete partition type 2. No surgical complication or difficulty was seen in any patient. All patients showed good hearing outcome postoperatively.* Conclusion*. Cochlear implantation could be a good treatment option for Waardenburg syndrome.

## 1. Introduction

 Waardenburg syndrome (WS) is a major cause of symptomatic sensorineural hearing loss (SNHL). It is an autosomal dominant disease characterized by dystopia canthorum, hyperplasia of the eyebrows, heterochromia iridis, white forelock, and congenital SNHL [1]. Clinically, WS is divided into 4 types based on the following clinical criteria [2]: the presence (type 1) or absence (type 2) of dystopia canthorum, additional upper limb anomalies and coarser facial characteristics (type 3), or Hirschsprung's disease (type 4). The genetic mutations differ among the types of WS (Table 1) [3].

The occurrence rates of SNHL in WS also differ among these types. About 60% of type 1 and type 3 children, and about 90% of type 2 and type 4 children, have SNHL [4]. While the hearing loss can be unilateral or bilateral and can vary in nature and severity, bilateral profound SNHL is the most common type of hearing loss.

Cochlear implantation (CI) is an option for patients with severe to profound bilateral hearing loss. However, few reports about CI in WS children have been published. The aim of this study is to describe the outcomes in five pediatric patients with WS who underwent CI at our institute.

## 2. Methods

A retrospective chart review was performed on patients who had undergone CI in the Otorhinolaryngology Department at the University of Tokyo Hospital from 1991 to 2014. Five patients were diagnosed as WS by their characteristic features or gene testing. Their clinical type, clinical course, preoperative hearing thresholds, high-resolution computed tomography (CT), and the course of hearing ability were evaluated. Meaningful auditory integration scales (MAIS), meaningful use of speech scale (MUSS), CI-2004 Japanese closed set three words' test, and 67-s monosyllable word tests were used to evaluate hearing performance after CI [5, 6].

## 3. Results

Table 2 shows the characteristics of the five children with WS who underwent CI. The average age at implantation was 2 years 11 months (ranging from 1 year 9 months to 6 years 3 months). Three patients were classified as type 1, one as type 2, and one as type 4. Four had congenital hearing loss and one had progressive hearing loss. The patient who had progressive hearing loss underwent CI at 6 years 3 months of age. Three patients (patients 3, 4, and 5) showed an autosomal-dominant pattern, and two patients (1 and 2) were sporadic.* PAX3* gene mutation was confirmed in one patient (4). Mutation was not detected in one patient (1). Three patients (2, 3, and 4) did not have gene testing. The average preoperative unaided threshold was 117.2 dB (105–135 dB) and the average aided threshold was 79.8 dB (60–84 dB). High-resolution CT of the temporal bone revealed that two patients (2 and 3) had incomplete partition type 2 while the other three patients had normal anatomy. No complication including a cerebrospinal fluid gusher during surgery was observed in any patient. All patients had full insertion of CI electrodes via scala tympani cochleostomy. Cefazolin or cefotiam was used as antibiotics for five days after surgery. No corticosteroids were used. CI24RE Contour Advance electrode was used for all patients. Facial nerve stimulation by CI was not seen in any of the patients.

**Figure 1:**
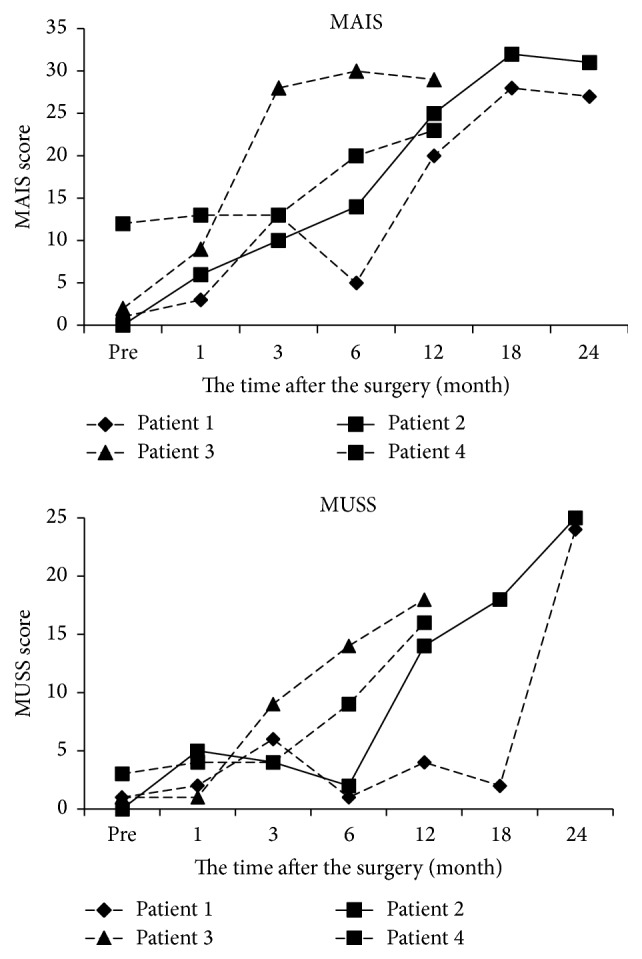
The results of MAIS and MUSS after CI.

Figure 1 shows the time course of MAIS and MUSS after the operation in four WS patients with congenital hearing loss. In all four patients, the MAIS scores increased immediately after CI and the MUSS scores rose slowly but surely.

Table 3 shows the results of postoperative thresholds and speech recognition tests. As it suggests, the average thresholds of cochlear implantation were below 40 dB for all patients. The average score of CI-2004 three words' tests is 78% and the score of 67-s monosyllable word tests in all three patients (1, 2, and 5) who could perform the tests improved to more than 80%.

## 4. Discussion

In this study, we reviewed five WS patients who underwent CI. One showed a progressive pattern of hearing loss and four had congenital hearing loss. Two had a cochlear malformation and the others displayed no anatomical anomalies. There was no difficulty or complication during the CI surgery. The postoperative hearing performance was generally good in all patients.

WS was first described by Waardenburg and is now classified into four types [1]. WS 1, which is the original type, is characterized by dystopia canthorum, abnormalities of pigmentation (white forelock, white eyelashes, leukoderma, and heterochromia iridis), and SNHL. WS 2 differs from WS 1 by the lack of dystopia canthorum. WS 3, also called Klein-Waardenburg syndrome, has upper limb abnormalities accompanied by the same characteristics as WS 1. WS 4, also known as Shah-Waardenburg syndrome, has Hirschsprung's disease with the features of WS 2 [2, 7, 8]. The prevalence of WS is estimated at approximately 1 patient per 42,000 individuals, and WS accounts for 1%–3% of patients of congenital deafness [2]. WS is mainly inherited in an autosomal-dominant pattern but some patients show an autosomal-recessive pattern. Several genetic mutations have been reported according to the classification. WS 1 and 3 have been linked to a mutation in the* PAX3* gene on chromosome 2q35 [9]. One subtype of WS 2 has a mutation in the* MITF* gene on chromosome 3p12.3–p14.1 [10]. In addition,* WS2B*,* WS2C*, and* SNAI2* mutations have an association with WS 2, and* EDNRB*,* EDN3*, and* SOX10* are considered to be a cause of WS 2 and WS 4 [11–14]. In these mutations,* EDNBR* and* SNAI2* mutations can be a cause of autosomal recessive HL. We encountered* PAX3* gene mutation in one patient (patient 4) with clinical subtype of WS 1. This patient showed autosomal dominant HL and was consistent with other reports. The other patient (patient 1) was evaluated for* MITF* mutation at another hospital and was negative. Considering that this patient showed phenotype 4, other genes such as* EDNRB*,* EDN3*, and* SOX10* should have been investigated.

In WS, some patients show a progressive hearing loss pattern. Of all reported patients with WS who underwent CI, including our four patients, only four of 46 (8.7%) involved progressive hearing loss. Although some studies did not clearly describe the WS type in those with progressive hearing loss, such patients are reportedly limited to WS type 2. Our patient with progressive hearing loss was also type 2, which was consistent with a previous report. Other reports have also suggested that WS type 2 involves progressive HL [15, 16]. Pingault et al. stated that the genetic findings in WS 2 and 4 are more complex than those in WS 1 and 3, and that WS 2 and 4 are genetically heterogeneous [3]. These genetic varieties may lead to various clinical features in WS 2 and 4, including progressive hearing loss.

Previous studies reported abnormal radiological findings in the cochlea in WS. Semicircular canal dysplasia, an enlarged vestibular aqueduct, and dysplasia of the cochlea have been reported [17, 18]; the malformations found in our patients were incomplete partition type 2. Abnormal histopathological studies include degeneration of the organ of Corti, stria vascularis, and saccular macula, but abnormalities of the bony architecture of the cochlea and labyrinth have not been reported [19]. Oysu et al. stated that the rate of temporal bone malformations in WS type 1 is lower than that of children with congenital hearing loss in general [17]. These data suggest that, in WS patients, severe cochlear abnormalities that can result in poor results with CI [20–22] are rare, and that good performance can be expected from a cochlear structural aspect.

The postoperative performance in our patients was generally good, which was consistent with previous reports [23–25]. El Bakkouri et al. [26] compared 30 WS patients with 85 patients with the* GJB2* mutation and reported no difference in CI performance. Miyagawa et al. [27] reported satisfactory auditory performance after CI in those where genetic mutations including two cases of WS had been detected. All of these reports indicate that WS patients are also good candidates for CI. In spite of these results, some factors must be considered. Pau et al. [24] reported that some patients with WS have auditory neuropathy and these patients attain less benefit from CI. Some studies reported that WS is related to behavioral disorders, with developmental or cognitive impairment [25, 28]. No such disorder was seen in our patients, but closer consideration should be given to whether patients have other disorders or impairments when deciding on CI for WS.

## 5. Conclusion

Five patients of CI in WS in our institute were reviewed. One showed a progressive pattern of hearing loss. Two showed cochlear malformations. There was no difficulty or complication during CI surgery. The postoperative performance was generally good in all patients. CI could be a good option for WS.

## Figures and Tables

**Table 1:** Classifications of Waardenburg syndrome.

	Clinical manifestations	The incidence rate of SNHL	Genetic mutation
Type 1	Dystopia canthorum, white forelock, white eyelashes, leukoderma, heterochromia iridis	60%	*PAX3*

Type 2	The absence of dystopia canthorum	90%	*MITF, WS2B, WS3B* *EDNRB, EDN3, SOX10* *SNAI2*

Type 3	Type 1 + upper limb abnormalities	60%	*PAX3*

Type 4	Type 2 + Hirschsprung's disease	90%	*EDNRB, EDN3, SOX10*

**Table 2:** Characteristics of the patients.

Patient number	Operation age	Clinical classification	The type of hearing loss	Anomalies of the inner ear	Genetic mutation	Hereditary form
1	2 y 3 mon	Type 4	Congenital	None	None^*∗*^	Sporadic
2	1 y 9 mon	Type 1	Congenital	IP2	N.A	Sporadic
3	2 y 2 mon	Type 1	Congenital	IP2	N.A	AD
4	2 y 2 mon	Type 1	Congenital	None	*PAX3*	AD
5	6 y 3 mon	Type 2	Progressive	None	N.A	AD

AD: autosomal dominant.

^*∗*^Patient one was not tested for all Waardenburg genes.

**Table 3:** Postoperative thresholds and speech recognition score.

	The average postoperative thresholds of CI	CI-2004 three words' test	67-s monosyllable words test
Patient 1	32.5 dB	70%	95%
Patient 2	23.8 dB	92%	100%
Patient 3	26.2 dB	75%	N.A
Patient 4	35.0 dB	75%	N.A
Patient 5	30.0 dB	N.A	80%
